# Cavernous Sinus Thrombosis Associated With Contralateral Pansinusitis: A Case Report

**DOI:** 10.7759/cureus.71192

**Published:** 2024-10-10

**Authors:** Daphne Gunness, Haris Duvnjak, Simon Morris, Huw Jones

**Affiliations:** 1 Ophthalmology, Wrexham Maelor Hospital, Wrexham, GBR; 2 Otolaryngology, Wrexham Maelor Hospital, Wrexham, GBR; 3 Otolaryngology, Betsi Cadwaladr University Health Board, Wrexham, GBR

**Keywords:** chronic rhino sinusitis, functional endoscopic sinus surgery (fess), peri-orbital cellulitis, pre-septal cellulitis, septic cavernous sinus thrombosis

## Abstract

A healthy woman in her 60s presented acutely with right-sided periorbital edema, erythema, neck pain, and fever. Initial examination and investigations suggested right-sided orbital cellulitis with contralateral pansinusitis. Despite treatment with IV antibiotics, her symptoms worsened, and inflammatory markers continued to rise. Further imaging revealed thrombosis of the right superior ophthalmic vein extending into the cavernous sinus and right jugular vein. She underwent urgent left functional endoscopic sinus surgery followed by long-term anticoagulation. After six months of anticoagulation, she remained free of morbidity, and repeat imaging confirmed the resolution of the thrombosis.

## Introduction

Orbital cellulitis is a common complication of sinusitis. According to Chandler’s classification, orbital infections include preseptal cellulitis, orbital cellulitis, subperiosteal abscess, orbital abscess, and cavernous sinus thrombosis (CST) [1]. This case report discusses the management of a patient who initially presented with symptoms indicative of orbital cellulitis and contralateral pansinusitis, which later revealed a more complex diagnosis of ophthalmic vein thrombosis and septic CST. Although rare, such cases can be life-threatening if left untreated, making early and appropriate management crucial. Clinicians should maintain a high index of suspicion and consider repeat imaging in cases that worsen despite optimal treatment.

## Case presentation

A female patient in her 60s presented with a one-day history of right-sided periorbital edema and erythema, accompanied by pain, fever, trismus, and right-sided neck discomfort. She reported experiencing left-sided facial pain for the past four weeks but denied any changes in vision, sore throat, dysphagia, or associated nasal symptoms. The patient was otherwise healthy, with no significant past medical history.

On examination, her temperature was 38.4 °C, while her heart rate, blood pressure, and respiratory rate remained within normal ranges. Initial assessments by the otolaryngology (ENT) and ophthalmology teams revealed right periocular edema and erythema, along with temporal chemosis. The best corrected visual acuity (BCVA) was 6/9 in the right eye and 6/5 in the left eye, with no prior recorded BCVA for comparison. The patient reported diplopia on dextroversion and dextrodepression, with associated restriction of eye movements observed. There was no loss of color vision, relevant afferent pupillary defect (RAPD), lagophthalmos, or proptosis, and the remaining cranial nerves were intact. Fundoscopic assessment was unremarkable, with no evidence of optic disc compromise. Otoscopy showed intact tympanic membranes bilaterally, and throat examination revealed symmetrical grade I tonsils. Flexible nasoendoscopy demonstrated left-sided nasal mucosal edema and mucopurulent discharge in the middle meatus.

Investigations

Non-contrast CT of the head and orbit, performed by the emergency department upon admission, revealed right-sided preseptal cellulitis without complicating features and left-sided pansinusitis, with no intraconal fat stranding or orbital abscess. Although the CT did not indicate any complicating features, a diagnosis of orbital cellulitis was made based on the examination findings.

Initial biochemical and hematological studies showed elevated inflammatory markers, with a CRP level of 246 mg/L and a WBC count of 26.4 × 10^9^/L.

Initial management

After 48 hours of IV co-amoxiclav 1.2 g three times daily, the patient reported improvement in eye pain and fever, along with a visible reduction in periocular edema and erythema. However, right-sided neck discomfort and trismus persisted, and right temporal chemosis and diplopia worsened. On examination, the BCVA remained unchanged, but right eye movements were restricted in all directions, which was confirmed by orthoptic assessment. Repeat biochemistry revealed an increased CRP level of 328 mg/L and a WBC count of 16.8 × 10^9^/L.

**Figure 1:**
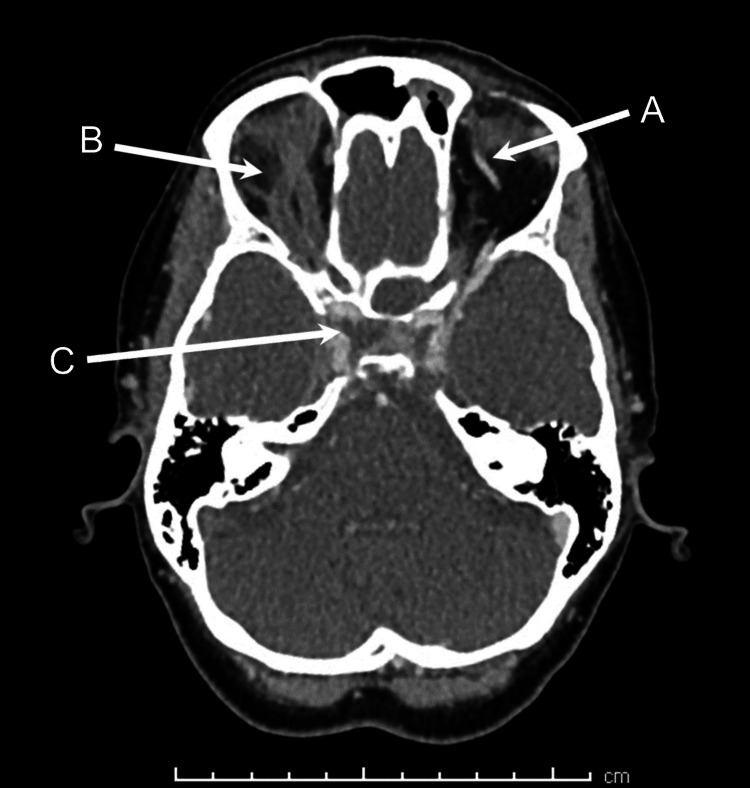
CT image showing the normal left ophthalmic vein (A), thrombosed right ophthalmic vein with retro-orbital fat stranding (B), and filling defect indicating right CST (C) CST, cavernous sinus thrombosis

**Figure 2:**
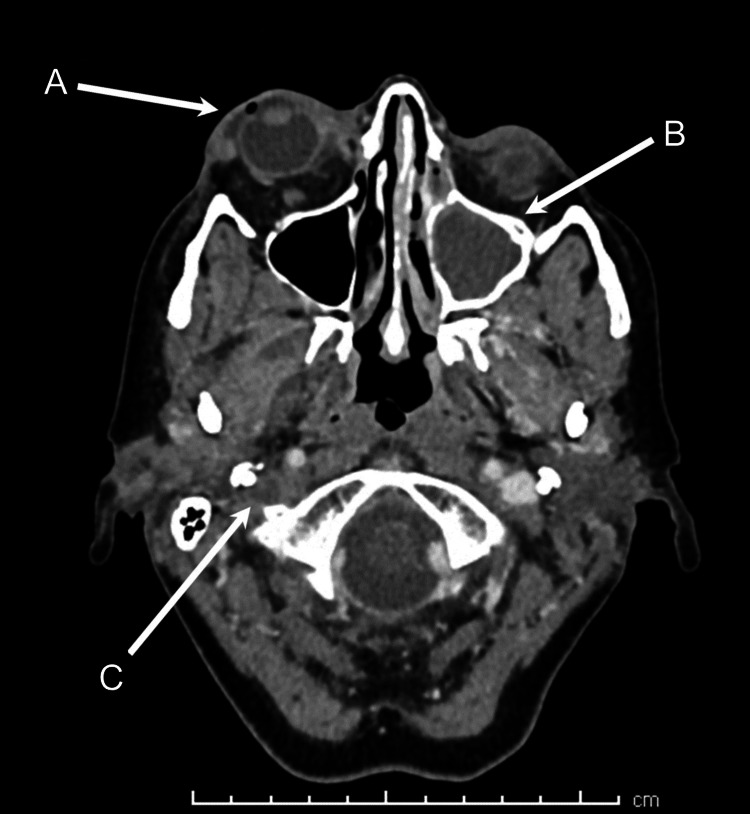
CT image showing right preseptal thickening with an associated gas pocket (A), further opacification of the left maxillary sinus (B), and extension of the thrombus into the right internal jugular vein (C)

**Figure 3:**
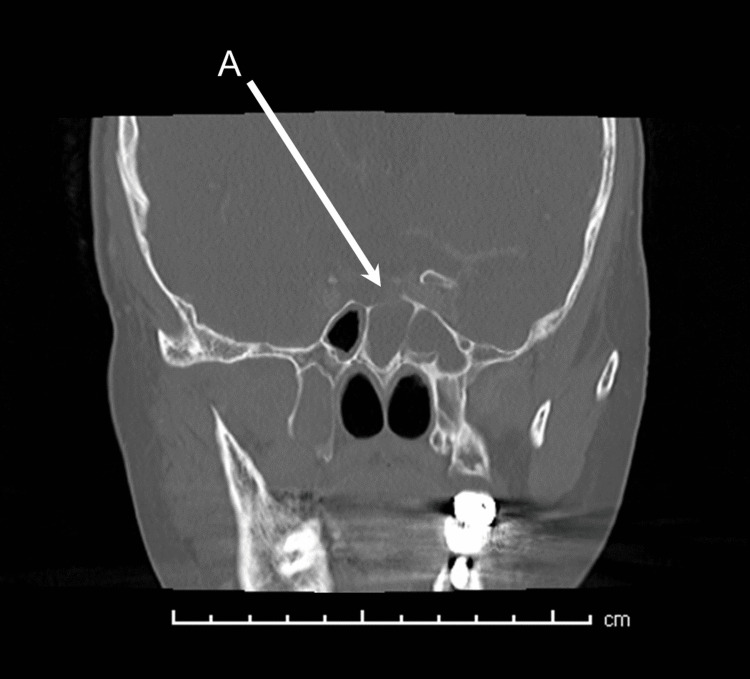
CT image showing opacified sphenoid sinus with accessory bony septations and a defect between the left side and the sella turcica (A)

In light of these worsening clinical features, imaging was repeated 48 hours after presentation. Contrast-enhanced CT of the head and orbit demonstrated right superior ophthalmic vein thrombosis, with right CST extending into the right jugular vein, sigmoid sinus, and lateral transverse sinus (Figure 1). Intraconal fat stranding behind the right globe was also observed, along with persistent thickening of the right preseptal tissues and an associated gas pocket (Figure 2). The opacification of the left paranasal sinus remained unchanged. It was noted that the left sphenoid sinus was septated, with a bony defect between the sinus and sella turcica (Figure 3). Changes were observed between the right lateral and medial pterygoid muscles, which were suspicious for an abscess; however, there were no features suggestive of an odontogenic etiology.

**Figure 4:**
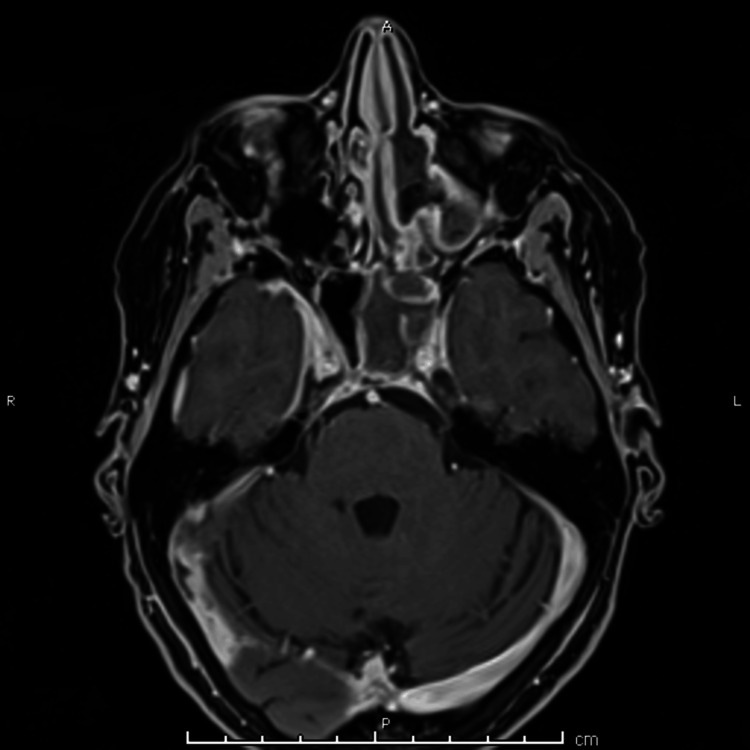
Axial view of MRI orbits with contrast showing left-sided ethmoid and sphenoid sinus opacification, consistent with findings from previous CT imaging

**Figure 5:**
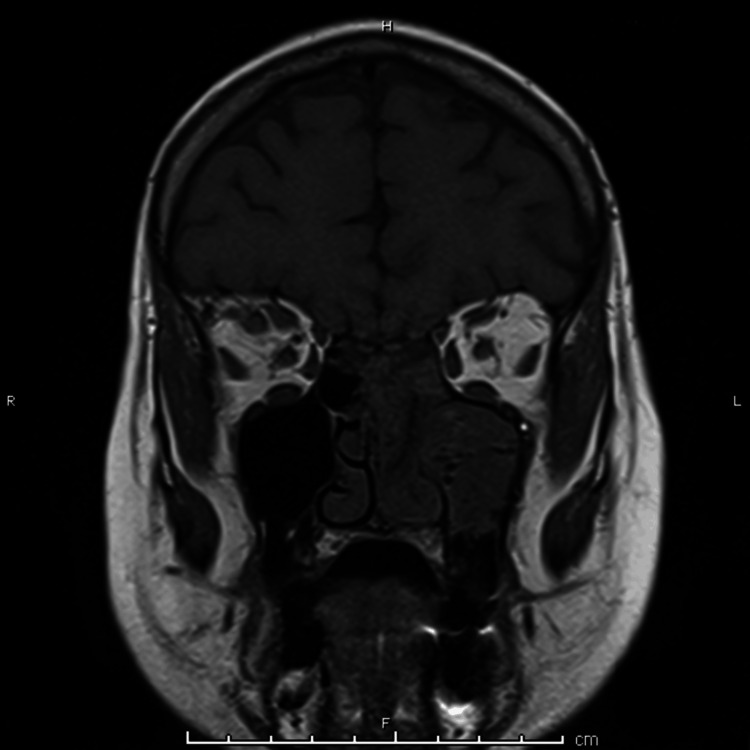
Coronal view of MRI orbits with contrast demonstrating sphenoid sinus opacification and right-sided orbital fat stranding

**Figure 6:**
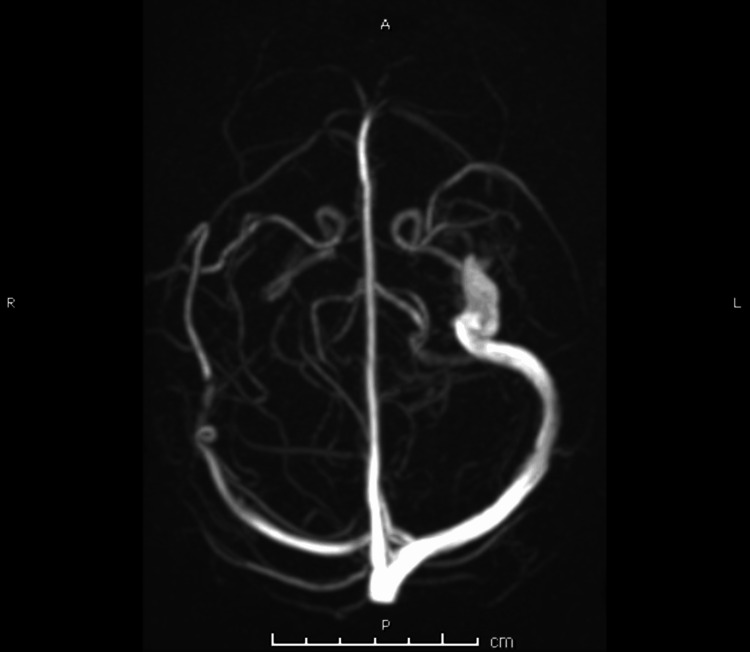
Axial view of 3D MR venogram showing right-sided thrombosis of the sigmoid sinus, transverse sinus, and cavernous sinus

An MRI of the head and neck with contrast was performed, confirming the extensive thrombosis (Figure 4, Figure 5, Figure 6).

Blood cultures taken on admission showed no growth after five days of incubation. A comprehensive set of hematological investigations was performed, including a coagulation screen, protein C and S levels, antithrombin levels, prothrombin mutation 20210 genotype, and factor V Leiden mutation. The results were negative, effectively excluding relevant pro-thrombotic conditions.

The oral and maxillofacial surgery team clinically ruled out a masticator abscess, attributing the trismus to localized soft tissue inflammation in the masticator space.

Differential diagnosis

Our initial differential diagnosis was orbital cellulitis secondary to sinusitis; however, this diagnosis did not account for the subsequent progressive symptoms of ophthalmoplegia, diplopia, and neck pain. Further investigation revealed thrombosis of the cavernous sinus and the ophthalmic vein.

Definitive management

IV ceftriaxone 2 g once daily and metronidazole 500 mg three times daily were initiated according to local trust guidelines. The patient was also prescribed daily saline nasal douching, fluticasone nasal spray, and xylometazoline nasal drops. Regular neurological observations were conducted. Upon identifying the extensive thrombosis, multidisciplinary input from otolaryngology (ENT), ophthalmology, neurology, hematology, microbiology, and oral-maxillofacial surgery was sought.

**Figure 7:**
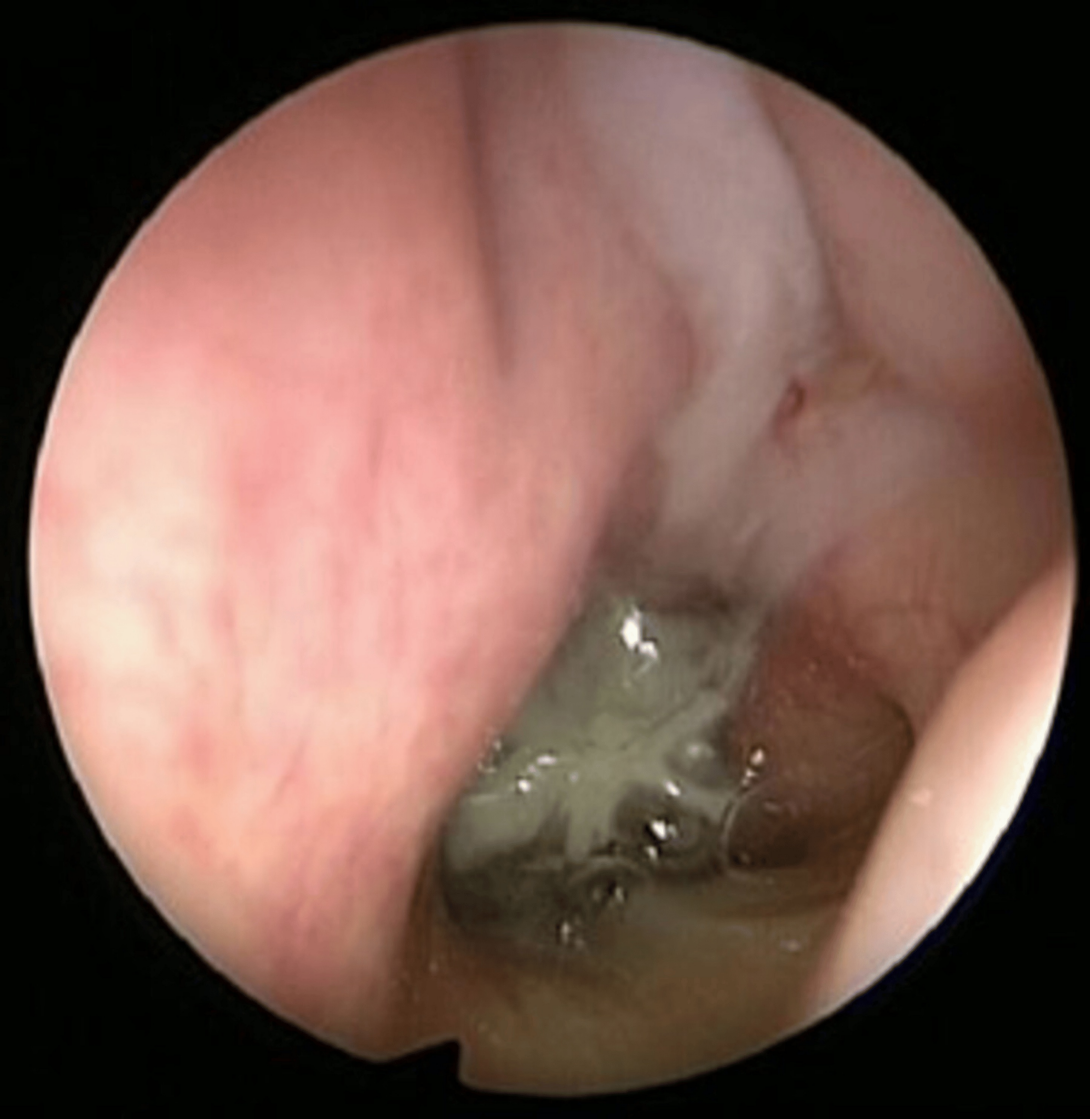
Endoscopic image showing mucopurulent discharge draining from the left middle meatus to the choanae, suggestive of rhinosinusitis

**Figure 8:**
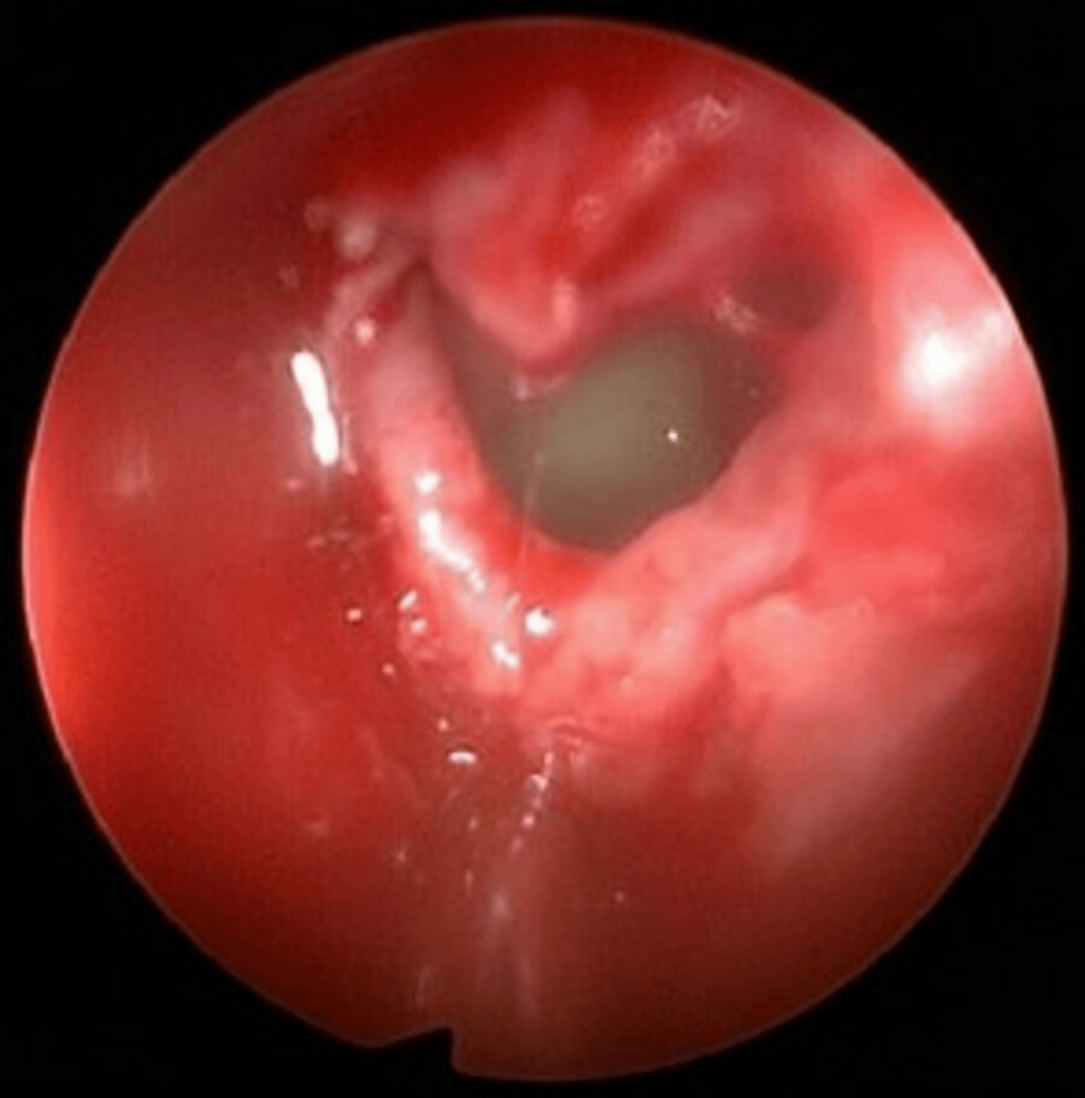
Endoscopic image showing mucopurulent discharge in the left posterior ethmoid sinus following ethmoidectomy

The ENT team performed functional endoscopic sinus surgery (FESS) with sphenoidectomy on day 3 of admission. Intraoperatively, persistent left-sided nasal mucosal edema was observed, accompanied by thick mucopurulent discharge draining from the left middle meatus into the posterior choanae (Figure 7). The surgical procedure included left uncinectomy, anterior ethmoidectomy, posterior ethmoidectomy, and sphenoidectomy with sinus washout (Figure 8). Pus swabs taken during the operation showed no growth after five days.

Enoxaparin 1 mg/kg twice daily was initiated to manage the thrombosis. Upon discharge, the patient was transitioned to warfarin, with a target INR of 2.5 (range 2-3) for a duration of six months.

Outcome and follow-up

After 14 days of IV antibiotics, the patient reported a complete resolution of symptoms. On examination, chemosis and periorbital signs had improved, with the BCVA of 6/6 in the right eye and 6/5 in the left eye. Serial orthoptic assessments demonstrated improvement in gaze restriction following treatment.

Upon discharge, she was advised to continue warfarin until her follow-up and serial imaging at a tertiary center. A three-month MRI indicated improvement in the thrombosis, and a six-month follow-up scan showed complete resolution, leading to the cessation of warfarin.

## Discussion

Orbital cellulitis is a common condition encountered by clinicians; however, septic CST is rare, with an estimated annual incidence of 0.2-1.6 per 100,000 people. Before the introduction of antibiotic therapy, septic CST was associated with a mortality rate approaching 100%. Although this has improved, the current mortality rate remains between 20% and 30% [2]. Septic CST can arise from any head and neck infection but is frequently associated with sinusitis. In cases of sphenoiditis, the infection can spread through osteomyelitis of the diploic bone or a bony deficit in the sphenoid, leading to thrombosis within the cavernous sinus, typically caused by *Staphylococcus aureus* organisms [3]. Neurological complications, which occur in approximately 50% of patients, depend on the extent of the thrombosis and may include abducens nerve palsy, meningitis, and stroke. This underscores the importance of prompt recognition and aggressive therapy by healthcare professionals [1].

The cavernous sinus is a venous plexus separated from the sphenoid sinus by the thin sphenoid bone [4]. The common signs of CST arise from the unique anatomy of the cavernous sinus and the cranial nerves it houses, particularly cranial nerves III to VI. The cavernous sinus receives venous drainage from multiple structures, including the superior ophthalmic veins and cerebral veins, ultimately draining into the internal jugular vein via the superior and inferior petrosal sinuses and sigmoid sinuses [5]. In our case report, the thrombus extended from the right superior ophthalmic vein through the right cavernous sinus into the right sigmoid sinus and transverse sinus.

Although clinical signs may raise suspicion of CST, the optimal diagnostic tests are CT venography and magnetic resonance venography [6]. These imaging modalities are highly sensitive and allow visualization of venous filling defects as well as potential etiologies of CST. The benefit of CT over MRI lies in its accessibility and cost-effectiveness. Non-contrast imaging may reveal indirect signs such as engorgement of the ophthalmic veins and exophthalmos but may miss the diagnosis. In our case, contrast-enhanced CT of the orbit identified unilateral enlargement of the ophthalmic vein and orbital fat stranding, along with contralateral sinusitis. Other radiological signs of CST include dilation and enhancement of the cavernous sinus with a convex lateral wall [7].

Chandler’s classification categorizes radiological findings of orbital infections into five types: type I (preseptal), type II (orbital cellulitis), type III (subperiosteal abscess), type IV (orbital abscess), and type V (CST) [1]. It is important to note that these stages do not necessarily progress in a linear fashion. In our case, the initial CT scan suggested type I; however, further imaging was consistent with type V. We therefore urge clinicians to remain vigilant for signs of progressive orbital cellulitis and maintain a low threshold for suspicion.

While there are consensus guidelines for cerebral venous thrombosis [7] and orbital cellulitis [8], no gold standard management pathway exists for septic CST. Expert opinion recommends a prolonged course of antimicrobial therapy, specifically anti-staphylococcal agents and metronidazole, alongside surgical intervention to control the source of infection, including FESS [3]. If there are no contraindications, anticoagulants are generally recommended for at least three months to prevent the progression of thrombosis.

A significant strength of this patient’s care was the access to and involvement of multidisciplinary teams throughout her journey. This included emergency physicians, ophthalmologists, head-and-neck radiologists, otolaryngologists, hematologists, neurologists, and orthoptists, as well as appropriate follow-up care for eye rehabilitation and neurology at a tertiary center.

A major challenge in this case was the extensive contralateral intracranial thrombosis resulting from left-sided sinusitis, which posed a diagnostic challenge due to the lateralization of symptoms. While two cases by Komatsu et al. [9] and Eweiss et al. [10] demonstrated contralateral sinus and cavernous sinus pathology, this is the first case, to our knowledge, to show such extensive ophthalmic and intracranial thrombosis. Septic CST typically presents with fever, headache, and eye swelling. Eye signs such as chemosis and proptosis are usually bilateral 90% of the time and are, in contrast to our case, red flags for intra-orbital pathologies and CST [3].

## Conclusions

Clinicians should maintain a low index of suspicion for septic CST in patients who do not improve, even when unilateral eye signs are present. Contrast-enhanced imaging should always be requested as an initial step, and clinicians should have a low threshold for repeating imaging in patients who fail to respond to optimal medical management. Currently, there is a lack of high-quality evidence guiding the management of septic CST. A multidisciplinary and patient-centered approach should be adopted when considering both short- and long-term management options, including anticoagulation.

## Appendices

Patient’s perspective

Initially, I thought I had a migraine. I took to my bed for three days as I felt dizzy, cold, and generally unwell. Then my right eye closed, and I could not open my jaw. I initially went to A&E with what I thought was a sinus infection. What was a complete surprise to me was that I had a large blood clot behind my right eye. I felt troubled by this, coupled with the lack of sleep and worry about the blood clot. On my release from the hospital, having reached the required INR blood level, I have had time to reflect at home. Now I have weekly INR checks, have given up all my physical activities, and generally am taking it very easy. It is difficult waiting for my next MRI scan at the 12-week point and what it might reveal. Life is both a little confusing and worrying, but I look ahead with confidence and again thank all the staff who I have met along the way so far.
